# Gut microbiome and metabolome signatures in calcium oxalate stone recurrence: a multi-omics study

**DOI:** 10.1186/s12934-026-02977-0

**Published:** 2026-03-10

**Authors:** Teng Cui, Yang Yang, Dirk Lange, Xiangyu Wang, Jimeng Ruan, Jiayuan Ji, Kai Dang, Yongan Zhou, Jing Xiao

**Affiliations:** 1 Department of Urology, Beijing Friendship Hospital, Capital Medical University, 95 Yong’an Road, Beijing, China; 2 Institute of Urology, Beijing Municipal Health Commission, Beijing, 100050 China; 3 Department of Urology, Xuanwu Hospital, Capital Medical University, 45 Changchun Street, Beijing, 100053 China; 4 The Stone Centre, Jack Bell Research Centre, Department of Urologic Sciences, M.H. Mohseni Institute of Urologic Sciences, University of British Columbia, 2660 Oak Street, Vancouver, BC V6H 3Z6 Canada

**Keywords:** Renal stone recurrence, *Escherichia-Shigella*, Caffeine metabolism, Metabolomics, Shotgun metagenomics, 16S rDNA gene sequencing

## Abstract

**Background:**

The incidence and recurrence rate of nephrolithiasis have been increasing annually. Recent evidence highlights a close association between the composition and function of the gut microbiome and the occurrence and recurrence of kidney stones. We performed a multi-omic study to investigate changes in gut microbiota and their metabolites during nephrolithiasis development and recurrence, and to explore the underlying molecular mechanisms. Stool samples from 37 recurrent stone patients, 38 first-episode stone patients, and 39 healthy controls were collected for 16S rDNA amplicon sequencing and liquid chromatography-mass spectrometry. Ten samples from each group were randomly selected for metagenomic sequencing.

**Results:**

Compared to incident cases, recurrent stone patients exhibited further reduced gut microbial richness and diversity, with enrichment of *Enterobacterales, Pseudomonadota, Gammaproteobacteria, Enterobacteraceae, Escherichia-Shigella*, and *Bacillia*. In the recurrent kidney stone group, 9 metabolites were upregulated and 86 downregulated, with enrichment of genes in purine and caffeine metabolism pathways. We identified 10 metabolites as recurrence biomarkers and significant correlations between *Escherichia-Shigella* and Asn-Tyr, Leu-Ala-Ile, Tyrosyl-Alanine, or 3'-hydroxyhexobarbital. Additionally, gender-specific gut microbiota signatures were observed. Oxalate decarboxylase and short-chain fatty acid-related enzymes decreased during stone formation but rebounded with recurrence. Caffeine and its metabolites were significantly downregulated in recurrent patients, suggesting a potential association with stone formation and recurrence that merits further investigation.

**Conclusions:**

Our study comprehensively characterizes gut microbiome and metabolome signatures associated with nephrolithiasis recurrence. The findings reveal that recurrent nephrolithiasis is characterized by impaired gut microbial evenness, enrichment of specific taxa including *Escherichia-Shigella*, and dysregulated metabolic pathways such as purine metabolism and caffeine metabolism. The 10 identified metabolites show promise as potential recurrence biomarkers, with notable correlations between *Escherichia-Shigella* and key metabolites. These results highlight the critical association of the gut microbiome-metabolome axis with renal stone recurrence, providing novel microbial and metabolic targets for early prediction, with potential implications for prevention and personalized treatment that require further validation.

**Supplementary Information:**

The online version contains supplementary material available at 10.1186/s12934-026-02977-0.

## Introduction

The global prevalence of kidney stones has reached alarming levels, with both incidence and recurrence rates demonstrating a concerning upward trend in recent years. Notably, this condition is increasingly affecting younger populations, imposing a substantial economic burden on healthcare systems worldwide [1, 2]. A recent population-based study revealed that the prevalence of kidney stones in China is 5.8% (6.5% in males and 5.1% in females), affecting approximately 1 in 17 individuals in the general population [3]. According to data from the National Health and Nutrition Examination Survey, approximately 22.4% of self-declared stone formers reported experiencing three or more episodes of stone passage [4]. Thus, developing new screening strategies and implementing early interventions may substantially enhance clinical outcomes for kidney stone patients.

Recent advances in 16S rDNA sequencing, metagenomics, and metabolomics have provided growing evidence for connections between kidney stone formation and microbial communities along with their metabolic byproducts. Emerging research suggests that the microbiome-metabolome axis may play a dual role in renal lithogenesis, serving as both promoters through direct/indirect mechanisms and potential inhibitors of stone development [5, 6]. Huang et al. [7] first characterized distinctive urinary microbiome-metabolome profiles and their correlations in nephrolithiasis patients. Burton et al. [8] identified multi-site microbiota dysbiosis as a hallmark feature of stone formers. Tasian et al. [9] demonstrated reduced gut microbial diversity in stone patients, particularly noting decreased abundances of butyrate-producing and oxalate-degrading bacteria, and Lange et al. [10, 11] reported that intestinal bacterial butyrate metabolism impairment—with reduced key biosynthesis enzymes and *Faecalibacterium prausnitzii*—is an oxalate-independent recurrent kidney stone disease risk factor, and noted gut and urinary microbiomes regulate nephrolithiasis via short-chain fatty acid-mediated intestinal barrier maintenance and oxalate degradation, with antibiotic-induced dysbiosis as a critical risk factor. Notably, a multi-species bacterial network centered on oxalate-metabolizing taxa has been identified as a key contributor to healthy oxalate homeostasis, with the absence of this cooperative microbial community further increasing recurrence susceptibility [12].

Miller et al. [13–15] identified 14 lithogenic metabolites shared by calcium oxalate and calcium phosphate stones, enriched in active stone formers’ urine to distinguish radiographic activity, and summarized the microbiome’s role in nephrolithiasis pathogenesis—including antibiotic-driven dysbiosis and microbe-metabolite interactions—and potential targeted therapies.

While existing research has extensively characterized omics profiles in nephrolithiasis formation, the microbiota-metabolome signatures of stone recurrence remain understudied. Critical gaps persist in direct comparisons between recurrent and first-episode patients [16, 17], with Ellison representing initial but limited exploration [18]. The objective of our study is to evaluate the microbiome and metabolome signals in patients with initial and recurrent kidney stones and to explore other associations related to the composition and diversity of the microbiome and metabolome in this population. To our knowledge, this is the first report utilizing a multi-omics approach to comprehensively analyze the microbiome and metabolome of gut microbiota in healthy individuals, first-episode patients and recurrent stone formers.

## Materials and methods

### Study population, study procedure and sample collection

Samples for this study were collected from patients undergoing surgery at the Urology Center of Beijing Friendship Hospital, affiliated with Capital Medical University, from July 2021 to April 2023, as well as from healthy individuals at the hospital's health examination center. A total of 1035 unilateral renal or ureteral calcium oxalate stone surgical patients were initially screened. After excluding ineligible individuals per supplementary criteria, 427 remained. Balanced matching of baseline characteristics including age, sex ratio, residence area, dietary structure, water intake and food intake identified 95 patients for 1-year follow-up, comprising 50 FS candidates and 45 RS candidates. During follow-up, 6 FS candidates were lost to follow-up, 4 were reclassified to RS, and 2 failed to meet enrollment criteria. Ultimately, the three groups compared consisted of 37 patients in the recurrent kidney stone (RS) group, 38 patients in the first-episode (FS) kidney stone group, and 39 patients in the non-stone (NS) group. Patients with unilateral kidney stones or unilateral ureteral stones enrolled before April 2023 were re-evaluated by imaging in April 2024. Patients were classified into the RS group if they self-reported ≥ 2 acute episodes of kidney or ureteral stones treated with medication or surgery, or if new stones or growth of existing stones (with any dimension increasing by at least 1 mm from baseline) were diagnosed by abdominal-pelvic CT, KUB X-ray, or urinary ultrasound one year after surgery. Otherwise, they were classified into the FS group (A single episode of unilateral kidney or ureteral stones, with no recurrence confirmed during the 1-year follow-up period). All patients in the RS and FS groups provided stone samples obtained during surgery or spontaneous passage. Stone component analysis was performed using Fourier-transform infrared spectroscopy (FTIR), the gold standard for stone typing. Stone types were classified as pure calcium oxalate (monohydrate or dihydrate) or mixed calcium oxalate, defined as calcium oxalate combined with calcium phosphate or carbonate apatite. Patients with uric acid stones, struvite stones, cystine stones or other rare stone types were excluded to ensure cohort homogeneity. Recent studies [19] indicate three key factors shaping gut microbiome composition—age, stone composition, and antibiotic residence—independent of urolithiasis status. Antibiotic use within 30 days, sex, hypertension, water intake, weight, and diet are also associated. To minimize confounding by factors known to shape gut microbiota composition, we prospectively matched the NS group to the RS and FS groups by age, gender ratio, dietary habits, BMI and residence, with balanced sample sizes (37–39 per group) to ensure adequate statistical power for multi-omics analyses. We included adults aged 18–75 who have lived in Beijing, Hebei and Tianjin for 3 years and meet the above inclusion criteria. Specific inclusion and exclusion criteria can be found in the supplementary information. Clinical characteristics recorded included age, gender, body mass index (BMI), history of stone episodes, family history, surgical events, clinical indicators (e.g., blood uric acid, creatinine, calcium, phosphate), and urinalysis parameters (e.g., urinary white blood cells count, red blood cells count, specific gravity, pH value). Urinary pH was measured immediately after voiding using a calibrated (daily calibration with standard buffers) pH meter.

During the 1-year follow-up, participants were further excluded if they developed conditions that met the initial exclusion criteria, including changes in dietary status, long-term antibiotic use (> 7 days), new-onset hypertension, diabetes, or malignancies.

Stool samples were collected from each participant during their consultations at the Urology Department of Beijing Friendship Hospital. All 114 stool samples were subjected to 16S rDNA sequencing and untargeted metabolomic analysis, and 10 samples were randomly selected from each of the three groups (30 samples in total) for shotgun metagenomic sequencing. Samples were immediately stored at -80 °C until analysis in 3 min. Written informed consent was obtained from all participants, and the study was approved by the Ethics Committee of Beijing Friendship Hospital (Ethics No. 2024-P2-433–01), adhering to the Declaration of Helsinki and CIOMS ethical guidelines.

### 16S rDNA microbial community profiling analyses

The hypervariable V4 region of the bacterial 16S rDNA gene was amplified using the degenerate PCR primers 515F (5'-GTGCCAGCMGCCGCGGTAA-3') and 806R (5'-GGACTACHVGGGTWTCTAAT-3'). The PCR amplification was performed in a 15 μL reaction system containing Phusion High-Fidelity PCR Master Mix, 0.2 μM each of forward and reverse primers and approximately 10 ng of template DNA. The thermal cycling protocol comprised an initial denaturation step at 98 °C for 1 min, followed by 30 cycles of: 98 °C for 10 s (denaturation), 50 °C for 30 s (annealing), and 72 °C for 30 s (extension), with a final extension at 72 °C for 5 min. PCR amplicons were purified using the magnetic bead purification method. Samples were mixed at equal density ratios by PCR amplicon concentration; after thorough mixing, PCR amplicons were detected and target bands recovered. The purification of PCR amplicons using the magnetic bead purification method involved mixing samples in equal density ratios based on the concentration of the PCR amplicons. After thorough mixing, the PCR amplicons were detected, and the target bands were recovered. After purification and quantification, the amplicons were pooled and sequenced using the NovaSeq 6000 (Illumina). The raw paired-end reads were assembled and subjected to quality filtering to obtain effective tags [20]. Denoising was performed using the DADA2 module in QIIME2 (202,202) to obtain the final Amplicon Sequence Variants (ASVs) [21]. To eliminate bias from uneven sequencing depth, all samples were rarefied to the minimum number of valid reads (65,000 reads per sample) using QIIME2 (202,202) before diversity analysis. Species annotation for each ASV was conducted using the Naive Bayes classifier in QIIME2 (202,202), with the Silva138.1 database.

Sequencing analysis included α-diversity analysis, β-diversity analysis and taxonomy analysis. The α-diversity analysis evaluated the diversity of taxa within individual samples. Alpha diversity was assessed in QIIME2 using seven metrics: Observed ASVs, Chao1, Shannon, Simpson, dominance, Good's coverage and Pielou's evenness. β-diversity analysis compared the differences in microbial composition among groups. Weighted and unweighted UniFrac distances were calculated using QIIME2 (202,202) and a heatmap was generated to display the UniFrac distances between samples, implemented in Perl. PICRUSt2 software was used to predict the functional profiles of the Kyoto Encyclopedia of Genes and Genomes (KEGG) and Clusters of Orthologous Groups (COG). Venn diagrams, ternary plots, and heatmaps at different taxonomic levels were generated using R (4.0.3), while petal plots, bar charts and phylogenetic trees were created in Perl (5.26.2).

### Untargeted metabolomics and analysis

Stool samples (0.5 g each) from three groups were placed in 1.5-mL microcentrifuge tubes (EP tubes). After adding 500 µL of 80% methanol, the samples were vortexed and centrifuged at 15,000 g, 4 °C for 20 min. The supernatant was diluted with liquid chromatography-mass spectrometry (LC–MS) grade water to achieve a final methanol concentration of 53%, then centrifuged again to collect the supernatant [22]. LC–MS analyses were performed by a Vanquish UHPLC system (Thermo Fisher, Germany) coupled with an Orbitrap Q ExactiveTM HF mass spectrometer or Orbitrap Q Exactive™ HF-X mass spectrometer (Thermo Fisher, Germany) in Novogene Co., Ltd. (Beijing, China). A quality control (QC) sample was prepared by mixing equal volumes of experimental samples. The QC sample was analyzed before, during and after the injection in the LC–MS/MS system to assess system stability throughout the experiment. Additionally, blank samples were included to eliminate background ions. The eluents used for both positive and negative polarity modes were as follows: eluent A consisted of 0.1% formic acid in water, while eluent B was methanol. The solvent gradient was configured in the following manner: 2% B, 1.5 min; 2–85% B, 3 min; 85–100% B, 10 min; 100–2% B, 10.1 min; 2% B, 12 min. The Q Exactive™ HF mass spectrometer operated in positive and negative polarity modes with a spray voltage of 3.5 kV, a sheath gas flow rate of 35 psi, a capillary temperature of 320 °C, an auxiliary gas flow rate of 10 L/min, and an S-lens RF level of 60. The auxiliary gas heater was set to 350 °C.

The mass spectrometry raw data collected by LC–MS/MS was imported into XCMS to perform peak extraction, peak quantification, peak alignment, compound identification and standardization processing. Compounds with a coefficient of variation (CV) greater than 30% in the QC sample relative peak area were excluded, resulting in the identification and relative quantification of metabolites. The identified metabolites were then annotated using the KEGG database (https://www.genome.jp/kegg/pathway.html), HMDB database (https://hmdb.ca/metabolites) and LIPIDMaps database (http://www.lipidmaps.org/). After converting the data using metaX (a flexible and comprehensive software for processing metabolomics data), partial least squares discriminant analysis (PLS-DA) was performed to obtain the variable importance in projection (VIP) for each metabolite [23]. Statistical significance (*P*-value) of the metabolites between the two groups was calculated based on t-tests, along with the fold change (FC) to assess the differences between groups. The thresholds for differential metabolite screening were set at VIP > 1.0, FC > 1.2 or FC < 0.833, with *P*-value < 0.05. Relevant graphs were generated using Python (3.5.0) and R (3.4.3).

### Shotgun metagenomics and analysis

Metagenomics is a method first proposed by Handelsman [24] to study the complete genomic information of microbial communities directly. We collected 10 samples from each group for metagenomic analysis. Total microbial DNA was extracted from stool samples using the Stool/Intestinal Content DNA Extraction Kit (Cat. No.: DP328, TIANGEN BIOTECH (BEIJING) CO., LTD.) per the manufacturer’s protocol with minor optimizations: 200 mg samples were homogenized in lysis buffer with glass beads, mechanically disrupted, digested with proteinase K, purified via phenol–chloroform extraction and silica membrane adsorption, and eluted in 50 μL elution buffer. DNA quality was validated by NanoDrop 2000 (A260/A280: 1.8–2.0; A260/A230: > 1.5) and 1% agarose gel electrophoresis.

Qualified DNA was subjected to library construction using the Rapid Plus DNA Lib Prep Kit for Illumina (Cat. No.: RK20208), followed by sequencing on the NovaSeq platform. Raw data from the NovaSeq sequencing platform were preprocessed using fastp (https://github.com/OpenGene/fastp) to obtain clean data for subsequent analyses. The clean data were assembled using MEGAHIT software [25, 26]. Following assembly, we conducted a series of analyses, including gene prediction, abundance analysis, species annotation and functional database annotation. This included abundance table generation at various taxonomic levels, statistical analysis of annotated gene counts, visualization of relative abundance, abundance clustering heatmaps, principal component analysis (PCA) and non-metric multidimensional scaling (NMDS) dimensionality reduction, ANOSIM analysis for inter- and intra-group differences based on functional abundance, metabolic pathway comparison and functional differential analysis using MetaGenomeSeq and linear discriminant analysis effect size (LEfSe).

### Statistical analysis

Continuous variables were expressed as the mean and standard deviation when normally distributed, and as the median with interquartile range (IQR) when not. The differences in continuous data between study groups were analyzed using the independent t-test, analysis of variance (ANOVA) and Kruskal–Wallis test, while the Chi-square test was employed for categorical variables. Python (3.5.0) and R (3.4.3) were used for various analyses and chart preparation. *P* < 0.05 was considered statistically significant. To control for false discoveries arising from multiple hypothesis testing in high-throughput data, p-values were adjusted using the Benjamini–Hochberg procedure to control the false discovery rate (FDR). Statistical significance after FDR correction was set at q < 0.05 (or FDR-adjusted *p* < 0.05). For analyses where specified, the raw *p*-values are also reported alongside FDR-adjusted values.

## Results

### Characteristics of study population

The study population was divided into three distinct groups: recurrent stone formers (RS group, n = 37), first-episode stone formers (FS group, n = 38) and non-stone subjects (NS-group, n = 39). The majority of stones in both the RS and FS groups were pure calcium oxalate stones, with a higher proportion of mixed calcium oxalate stones in the RS group (13.51% vs. 10.53% in the FS group) (Table S1). No significant differences were observed in terms of age (*P* = 0.824), gender (*P* = 0.855) or Scr (*P* = 0.108) among the three groups. Although BMI and Ca were elevated in the RS group, no statistically significant intergroup difference was observed (*P* = 0.490, 0.176). The USG levels were higher in both the FS group (*P* = 0.026) and RS group (*P* = 0.001) than in the NS group (Table 1).

**Table 1:** Comparison of general characteristics among the RS group, the FS group and the NS group

Variables	RS group (n = 37)	FS group (n = 38)	NS group (n = 39)	*P* value
Age (yr, mean ± SD)	47.62 ± 10.79	46.16 ± 8.82	47.28 ± 12.16	0.8^*^
Gender (%)				0.9^※^
Male	25 (67.57)	24 (63.16)	25 (64.12)	
Female	12 (32.43)	14 (36.84)	14 (35.90)	
BMI (kg/m^2^, mean ± SD)	27.21 ± 4.31	26.01 ± 3.11	25.68 ± 2.56	0.5^*^
Family History(%)				**0.05**^※^
Yes	7 (18.92)	3 (7.89)	3 (7.69)	
No	30 (81.08)	35 (92.11)	36 (92.31)	
Operating Time (min, mean ± SD)	71.52 ± 40.27	54.97 ± 22.77	NA	**0.03**^#^
Average CT Value (HU, mean ± SD)	589.89 ± 222.74	527.50 ± 269.59	NA	0.2^*^
Serum Uric Acid(μmol/L,mean ± SD)	395.84 ± 80.36	356.11 ± 79.40	328.56 ± 86.57	**0.002**^#^
P(mmol/l, mean ± SD)	1.120 ± 0.16	1.15 ± 0.23	1.23 ± 0.14	0.01^*^
Ca (mmol/l, mean ± SD)	2.29 ± 0.08	2.31 ± 0.09	2.32 ± 0.11	0.2^#^
Scr (umol/L, mean ± SD)	83.83 ± 21.39	82.51 ± 27.11	73.20 ± 15.03	0.1^*^
Urine PH (mean ± SD）	6.43 ± 0.69	6.05 ± 0.70	6.21 ± 0.52	0.009^*^
USG (mean ± SD）	1.016 ± 0.004	1.018 ± 0.006	1.021 ± 0.007	0.001^*^

Bold values indicate statistically significant differences between groups (P < 0.05). Comparisons were made using Kruskal–Wallis test(*), Analysis of Variance(ANOVA,#), and 2-sided Chi-square test(※). BMI, body mass index; NA, not applicable.; USG, urinary specific gravity; Scr, serum creatinine;

The FS group exhibited elevated serum uric acid (395.84 vs 328.56 mg/dL, *P* = 0.002) and reduced phosphate levels (1.15 vs 1.26 mg/dL, *P* = 0.01) versus controls. The RS group demonstrated higher urinary pH (6.43 vs 6.05, *P* = 0.007) and significant familial predisposition compared to controls. Both stone cohorts (RS and FS groups) showed decreased urine specific gravity (1.015, 1.018 vs 1.023 controls, *P* < 0.05) (Table S2).

### Microbial diversity and composition analysis based on 16S rDNA sequencing

**Fig. 1:**
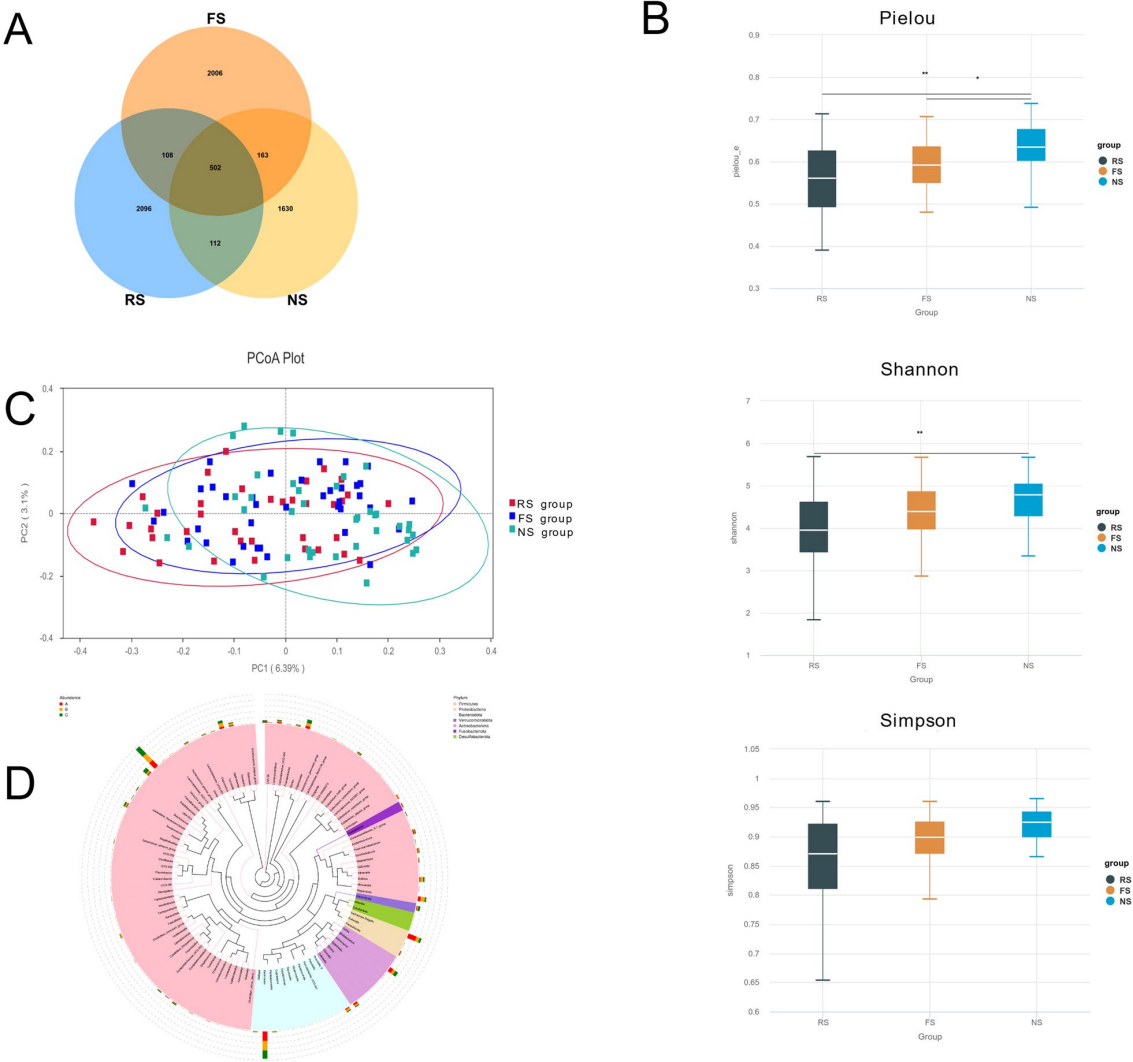
Gut microbiome diversity and structure analysis. **A** Venn diagram showing the number of unique and shared Amplicon Sequence Variants (ASVs) among the RS, FS and NS groups. **B** Boxplots of α-diversity indices (Shannon, Simpson, and Pielou's evenness) among the three groups. Statistical differences were determined using the Kruskal–Wallis test, with significance set at *P* < 0.05. **C** PcoA based on weighted and unweighted UniFrac distance among three groups. Intergroup differences in microbial composition were verified by ANOSIM (*P* < 0.05). **D** Cladogram presenting phylogenetic distribution of microbiota at phylum, class, order, family, and genus levels among three groups

Following rigorous quality control processing, a total of 8,671,825 high-fidelity sequencing reads were retained. Subsequent taxonomic classification identified 6,796 ASVs. The Venn diagram (Fig. 1A) identified 1,630 unique features in the NS group, 2,006 in the FS group, and 2,096 in the RS group, with 502 conserved features common to all groups. The rarefaction curves, rank-abundance distributions and species accumulation curves demonstrated that there was sufficient sample size and sequencing depth for subsequent analysis (Fig. S1).

Alpha diversity via Kruskal–Wallis test showed that gut microbial diversity (Shannon, Simpson, Pielou indices) was significantly lower in stone patients (RS and FS groups) than in the NS group (Fig. 1B), while richness (Chao1, Observed species) showed no intergroup differences. RS and FS groups exhibited notable comparative trends: Shannon index was numerically lower in RS than in FS (*P* = 0.067), and Simpson, Pielou indices showed no significant RS-FS differences (*P* = 0.259, 0.156 respectively) but stronger divergence from NS in RS (Simpson: *P* = 7.000 × 10⁻^4^; Pielou: *P* = 2.000 × 10⁻^4^) than in FS (Simpson: P = 0.021; Pielou: *P* = 0.015). PcoA at the ASV level revealed distinct microbiota compositions among NS, FS and RS groups (Fig. 1C), confirmed by ANOSIM test (Table S3-4). Sequences from all samples were categorized into seven taxonomic classification levels, including two kingdoms, 20 phyla, 40 classes, 74 orders, 121 families, 287 genera and 198 species. A phylogenetic tree was constructed using the ASVs (Fig. 1D).

**Fig. 2:**
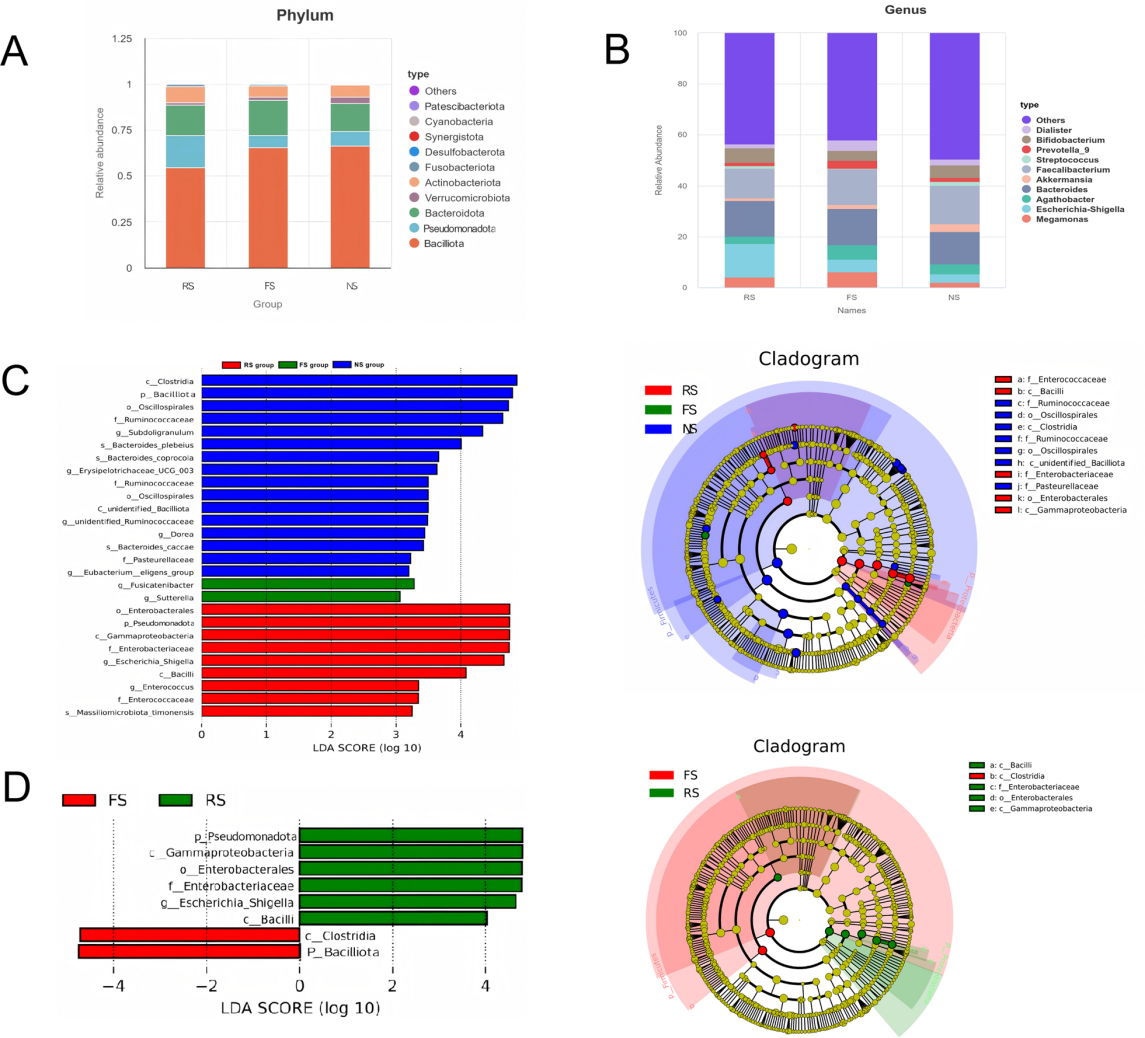
Microbial differences among RS, FS, and NS groups. **A** Stacked bar charts showing the relative abundances of bacterial phyla among the three groups. **B** Bar charts displaying the relative abundances of dominant bacterial genera among the three groups. **C **Difference in abundance estimated by LEfSe among RS, FS and NS groups and a taxonomic cladogram was presented (LDA score > 3.0, *P* < 0.05; Kruskal–Wallis test followed by pairwise Wilcoxon rank-sum test). **D** Difference in abundance estimated by LEfSe between the RS group and the FS group and a taxonomic cladogram was presented(LDA score > 4.0, *P* < 0.05; Wilcoxon rank-sum test)

At the phylum level, *Bacilliota*, *Pseudomonadota*, *Bacteroidota* and *Actinobacteriota* were the four phyla with the highest relative abundance in the three groups (Fig. 2A). The relative abundance of *Pseudomonadota* was significantly higher in RS (17.44%) than in FS (6.50%). Consistently, at the class level, *Gammaproteobacteria* (a major class within *Pseudomonadota*) also showed a significantly higher relative abundance in RS (17.44%) compared to FS (6.50%). In contrast, *Clostridia* (class level) and *Subdoligranulum* (genus level) were less abundant in RS (40.10% and 1.98%, respectively) than in FS (50.07% and 6.32%, respectively). All differences were analyzed by t-test with Benjamini–Hochberg FDR correction, and considered significant at q < 0.05. At the genus level, *Pseudomonadota* was the genus (Fig. 2B) with the highest relative abundance in the three groups (14.12%, 14.40% and 12.49%).In order to explore more differences in the flora, we performed Lefse analysis.

To study differences in flora, LEfSe analysis (LDA > 3.0, *P* < 0.05) was performed and identified distinct microbial signatures across groups (Fig. 2C). Recurrent stone patients exhibited enrichment of *Enterobacterales, Pseudomonadota, Gammaproteobacteria, Enterobacteraceae, Escherichia-Shigella, Bacillia, Enterococcus, Enterococcaceae, Massiliomicrobiota_timonensis*. First-episode stone patients showed increased abundance of *Fusicatenibacter* and *Sutterella*, while non-stone controls were enriched in *Clostridia, Bacilliota, Oscillospirales, Ruminococcaceae* and *Subdoligranulum*. Individual group comparisons further confirmed RS enrichment of *Pseudomonadota, Gammaproteobacteria, Enterobacteraceae, Enterobacteriaceae, Escherichia-Shigella* and *Bacillia*, whereas the FS group uniquely harbored *Clostridia* and *Bacilliota* (Fig. 2D). These taxonomic profiles were validated by metagenomic species-level analysis, demonstrating congruent enrichment patterns across methodologies.

### Functional profiling of gut microbiota via shotgun metagenomics

**Fig. 3:**
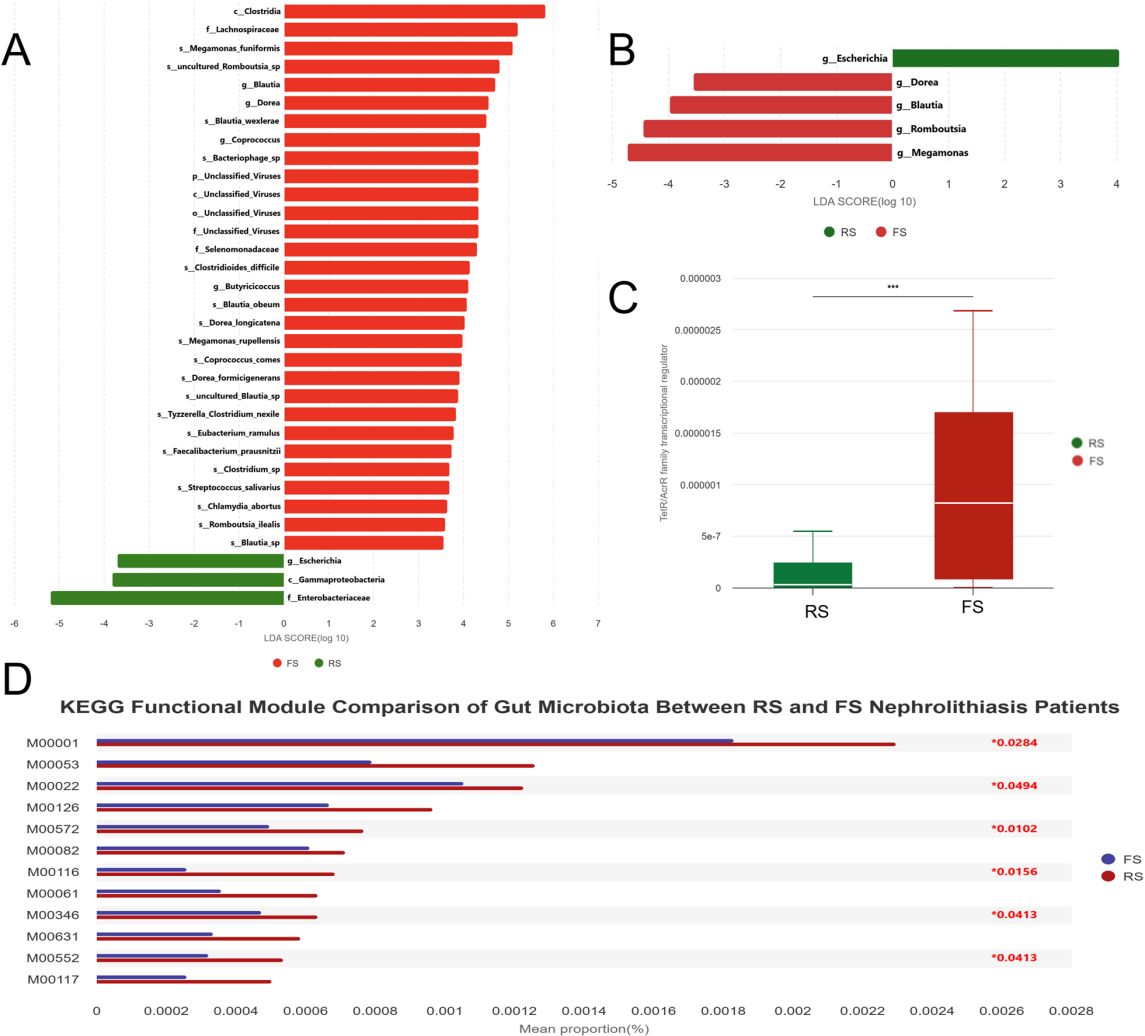
Functional and taxonomic characteristics of gut microbiota between RS and FS groups. **A**,** B** LEfSe analysis identifying differentially abundant taxa between RS and FS (LDA score > 3.5, *P* < 0.05; Wilcoxon rank-sum test). **C** Boxplot of TetR/AcrR family transcriptional regulator gene abundance differences between RS and FS Groups. **D** KEGG functional module comparison of gut microbiota between RS and FS nephrolithiasis patients. Differences were analyzed by MetaGenomeSeq, with FDR-adjusted *P* < 0.05 considered significant (marked with *)

The 10 patients randomly selected from each group for metagenomic sequencing had consistent demographic characteristics with the remaining patients in the corresponding group, ensuring no significant bias was introduced by the sampling strategy(Table S5). The overall taxonomic features of the gut microbiome were largely consistent with those from 16S rDNA sequencing(Fig. 3A, B). Key genes involved in oxalate degradation in the gut microbiota of calcium oxalate stone patients centered on oxalate: formate antiporter (OxlT), formyl-CoA transferase (frc) and oxalyl-CoA decarboxylase (oxc), supplemented by other metabolic pathway genes such as oxalate decarboxylase (oxdc) and short-chain fatty acid (SCFA) synthesis genes. In our study, the gene abundances of frc and oxc showed no differences between the recurrent and first-episode stone patients. However, we found that the gene abundance of oxdc, another key oxalate-degrading enzyme, was decreased in FSpatients but increased in RS stone patients, with a statistically significant difference between the two groups (*P* = 0.017). Additionally, MetaGenomeSeq analysis identified a significant difference in the abundance of the TetR/AcrR family transcriptional regulator gene (K22106) between the RS and FS groups (*P* = 0.038) (Fig. 3C).

Key enzymes related to SCFA production, such as butyrate kinase, acetyl-CoA acetyltransferase, acyl-CoA synthetase and β-ketoacyl-ACP synthase, also showed descriptive differences in abundance between FS and RS groups—with lower levels in the FS group and relatively higher levels in the RS group—but these changes did not reach statistical significance (all FDR-adjusted *P* > 0.05). These observations are exploratory and lack sufficient statistical support to infer potential biological relevance to stone recurrence, requiring validation in larger, well-powered cohorts. Functionally, multi-group difference analysis identified distinct differences in KEGG modules among the three groups associated with calcium oxalate stone formation and recurrence, including those involved in glycolysis (M00001), purine nucleotide biosynthesis (M00053), fatty acid biosynthesis (M00082), cholesterol biosynthesis (M00572), glyceride degradation (M00126), methane metabolism (M00116), D-glucuronate degradation (M00061), formaldehyde assimilation (M00346), trehalose biosynthesis (M00631), GDP-mannose biosynthesis (M00552), the tricarboxylic acid (TCA) cycle (M00117), and β-galactoside degradation (M00022) (Fig. 3D).

### Analysis of differential metabolites and their correlation with microbial abundance

Metabolomic analysis identified a total of 1,562 and plotted using KEGG metabolic pathways, such as Amino acid metabolism, Lipid metabolism and Carbohydrate metabolism (Fig. S2).

**Fig. 4:**
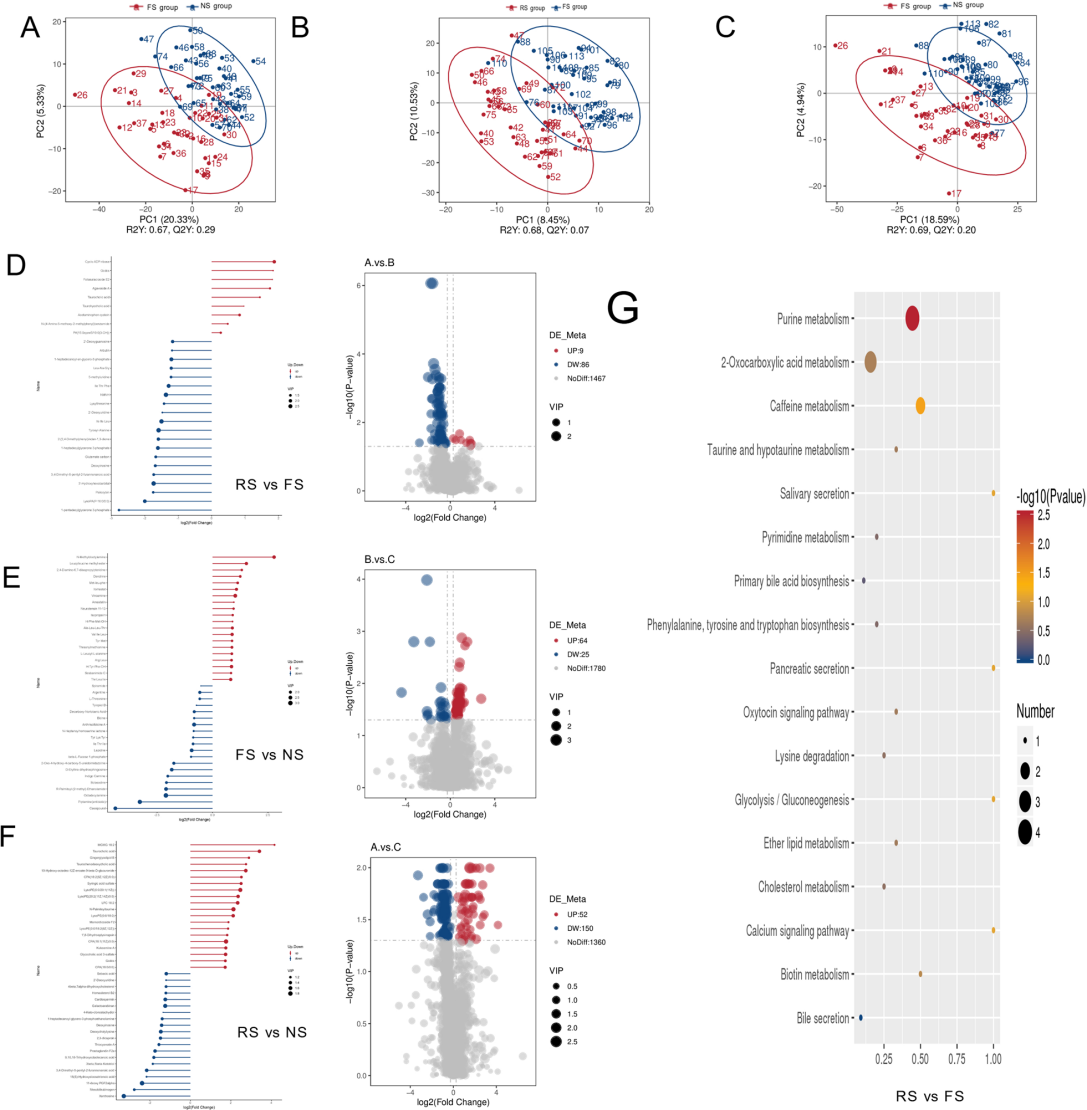
Differentially expressed metabolites among three groups. Red dots represent up-regulated metabolites, blue dots represent down-regulated metabolites. **A** PLS-DA of negatively charged metabolites between the RS group and the FS group(R^2^Y = 0.68, Q^2^Y = 0.07). **B** PLS-DA of negatively charged metabolites between the RS group and the FS group(R^2^Y = 0.67, Q^2^Y = 0.29). **C** PLS-DA of negatively charged metabolites between the RS group and the NS group(R^2^Y = 0.69, Q^2^Y = 0.20). **D** Volcano plot of DEMs between RS and FS, with thresholds set at VIP ≥ 1.0, fold change (FC) ≥ 1.2 or ≤ 0.835, and *P* < 0.05 (t-test). **E** Volcano plot of DEMs between FS and NS, with thresholds set at VIP ≥ 1.0, FC ≥ 1.2 or ≤ 0.835, and *P* < 0.05 (t-test). **F** Volcano plot of DEMs between RS and NS, with thresholds set at VIP ≥ 1.0, FC ≥ 1.2 or ≤ 0.835, and *P* < 0.05 (t-test). **G** KEGG enrichment analysis of the DEMs between RS and FS. The y-axis shows -log10(P-value), with P-values calculated using hypergeometric test (*P* < 0.05)

PLS-DA analysis distinguished the RS, FS and NS groups (Fig. 4A–C), indicating differences in the metabolism of gut microbiota among the three groups. Comparing the RS and FS groups, 9 metabolites were upregulated and 86 downregulated in the RS group. Key upregulated metabolites included Foliasalacioside E2(FC = 3.519, *P* = 0.050), Gcdcs(FC = 3.578, *P* = 0.039), and cyclic ADP-ribose(FC = 3.66, *P* = 0.043), whereas major downregulated metabolites were LysoPA(P-16:0/0:0) (FC = 0.250, P < 0.001), Psilocybin(FC = 0.298, P < 0.001), 1-pentadecylglycerone 3-phosphate(FC = 0.146, *P* = 0.039) and 3'-Hydroxyhexobarbital(FC = 0.300, *P* = 8.57E-07) (Fig. 4D). 64 metabolites were significantly up-regulated (VIP > 1.0, FC > 1.2 or FC < 0.833 and *P* value < 0.05) in the FS group compared to the NS group, N-Methyldioctylamine (FC = 6.843, P = 0.020), Leucylleucine methyl ester (FC = 2.881, *P* = 0.002) and 2,4-Diamino-6,7-diisopropylpteridine (FC = 2.496, *P* = 0.002) were most significantly elevated. Meanwhile, 25 metabolites were significantly reduced, especially Cassipourol (FC = 0.0481, *P* = 0.015) and Piptamine(antibiotic) (FC = 0.103, *P* = 0.002) (Fig. 4E). Compared with the NS group, the RS group had 52 upregulated metabolites, with MGMG 18:2 (FC = 17.780, *P* = 0.036), Taurocholic acid (FC = 10.605, *P* = 0.010), and Gingerglycolipid B (FC = 7.414, *P* = 0.028) showing the most significant elevations. Meanwhile, 150 metabolites were significantly downregulated in the RS group, particularly 11-deoxy PGF2α (FC = 0.191, *P* = 0.028), Mesobilirubinogen (FC = 0.146, *P* = 0.019), and Xanthosine (FC = 0.102, *P* = 0.012) (Fig. 4F).Additionally, KEGG pathway analysis of the differentially expressed metabolites (DEMs) between the RS group and the FS group revealed significant enrichment in Purine metabolism(*P* = 0.003) and Caffeine metabolism (*P* = 0.035) (Fig. 4G).

We hypothesized that the levels of these metabolites could be associated with the relative abundance of bacterial taxa identified in our study. We computed a correlation matrix between metabolites and species abundances (Fig. 6A), which revealed strong correlations for many of the taxon-metabolite pairs.

### Identification of potential recurrence biomarkers and microbiota-metabolite associations

**Fig. 5:**
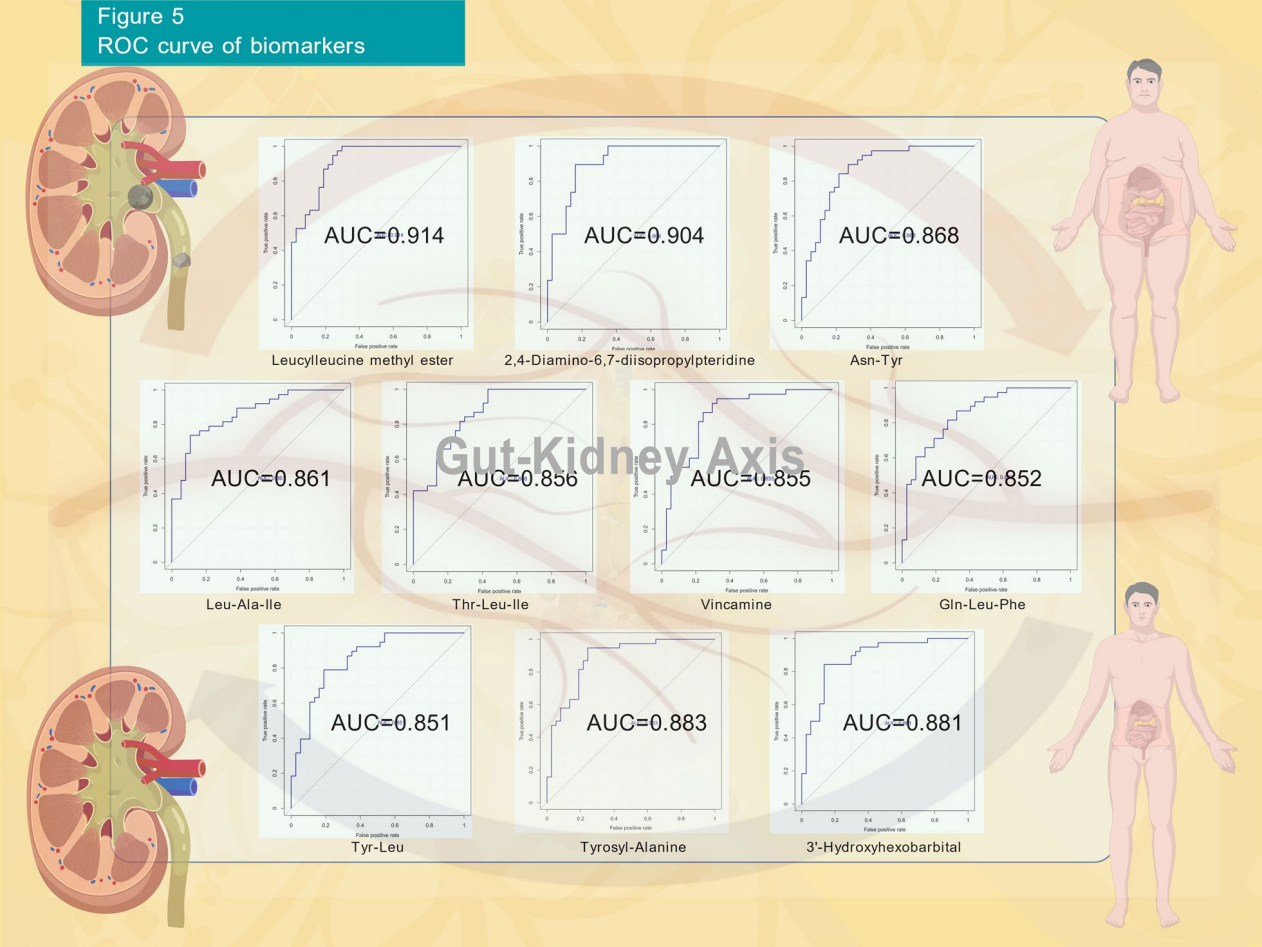
ROC curve of biomarkers. This figure depicts the ROC curves and AUC areas of 10 selected biomarkers in positive and negative modes. The discriminative power of each biomarker is evaluated by AUC value (ranging from 0.851 to 0.914), with higher AUC indicating better predictive performance for kidney stone recurrence

**Fig. 6:**
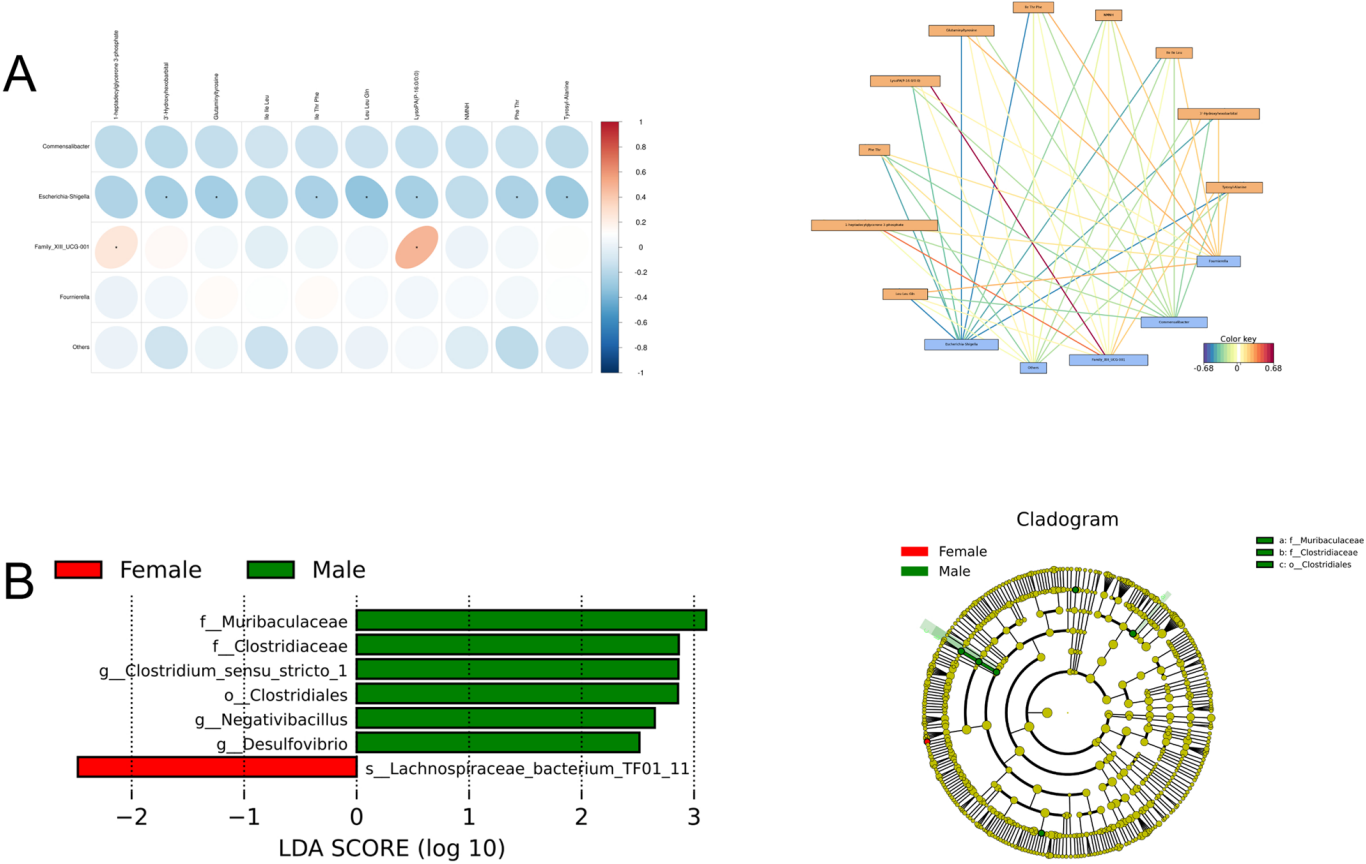
Correlation diagram of differential metabolites and microbial communities between the RS group and the FS group, and gender-based analysis of differential microbial communities. **A** Correlation heatmap and correlation network diagram analysis of differentially expressed metabolites and differentially abundant microbiota. The yellow box represents metabolites, and the blue box represents bacterial genera; The color of the line represents correlation, red represents positive correlation, and blue represents negative correlation. Statistical analysis was performed using Spearman’s rank correlation test, with significance set at *P* < 0.05. **B** Difference in abundance estimated by LEfSe between the male and female patients. Statistical analysis was performed using Wilcoxon rank-sum test, with LDA score > 2.0 and *P* < 0.05 considered significant

We evaluated the predictive capability of DEMs for kidney stone recurrence using ROC analysis. The results revealed by integrating positive and negative modes indicated that 10 DEMs, including Leucylleucine methyl ester, 2,4-Diamino-6,7-diisopropylpteridine, Asn-Tyr, Leu-Ala-Ile, Thr-Leu-Ile, Vincamine, Gln-Leu-Phe, Tyr-Leu, Tyrosyl-Alanine and 3'-Hydroxyhexobarbital, exhibited AUC values greater than 0.85, indicating good discriminative power. (Fig. 5) These 10 metabolites were identified through comparison between the RS group and the FS group, all showing a downregulation trend in the RS group. Furthermore, we found significant correlations between *Escherichia-Shigella* and Asn-Tyr, Leu-Ala-Ile, Tyrosyl-Alanine and 3'-Hydroxyhexobarbital (Fig. 6A).

### Gender-based analysis of gut microbiota composition

We conducted diversity analysis and LefSe analysis on the gut microbiota of male (n = 49) and female (n = 26) participants in the RS and FS groups and significant statistical differences were observed between the two groups. The results showed that the Simpson, Shannon and Chao1 indices of gut microbiota in male patients were lower than those in females. LEfSe analysis showed *Muribaculaceae, clostridiaceae, Clostridiun-*sensu*-stricto, Clostridiales, Negativibacillus* and *Desulfovibrio* were enriched in male patients. The relative abundance of *Lachnospiraceae bacteria TF01-11* was higher in females than in males. It may be related to gender differences in kidney stones (Fig. 6B).

## Discussion

Recent studies increasingly highlight a close association between gut microbiota and renal stone formation and recurrence [27, 28]. Previous studies show clinical factors like BMI, blood calcium, alcohol intake, hypertension, and diabetes are linked to renal stone formation [29, 30]. In our research, stone patients differed significantly from healthy individuals in urine specific gravity, serum uric acid, family history, and urinary acidity. Urine specific gravity varied notably among the three groups, with stone patients having lower levels, possibly linked to water intake awareness. Kidney stone patients who have experienced renal colic usually consume more water during hospitalization. The RS group showed more alkaline urine (pH > 6.5, 43.24%) than FS (25.64%) and NS (23.68%) groups, indicating a trend in recurrent stone patients. This trend may be attributed to the elevated incidence of urinary tract infections and the subsequent formation of infection-related stones among recurrent patients [31]. The RS group had a slightly higher proportion of mixed calcium oxalate stones (13.51%), which may be related to repeated urinary tract inflammation or persistent metabolic disturbances in recurrent patients—consistent with our earlier observation of higher urinary pH in the RS group.

Alpha diversity analysis showed a more diverse gut microbiome characterized by significantly lower evenness and dominance in stone patients than in the NS group, with no significant differences in richness as reflected by Chao1 and Observed species. RS and FS comparisons highlight recurrence-related microbial shifts: the numerical reduction of Shannon index in RS (*P* = 0.067) suggests a trend of further diversity loss in recurrent cases. While Simpson and Pielou indices show no significant RS-FS differences, their more pronounced divergence from NS in RS indicates deeper imbalance. These findings indicate persistently reduced evenness and further enriched dominant taxa, not species number changes, are key microbial shifts driving recurrence. They consolidate harmful bacteria dominance and exacerbate metabolic dysregulation linked to stone recurrence. A comparative analysis among the RS, FS and NS groups revealed that gut microbiota associated with renal stone formation and recurrence. We observed a sequential and gradual increase in the relative abundance of *Escherichia-Shigella* across the three study groups, with the lowest levels in the NS group, intermediate levels in the FS group, and the highest levels in the RS group. This stepwise elevation was significantly associated with the frequency of stone episodes, suggesting a potential cumulative effect of *Escherichia-Shigella* enrichment in stone formation and recurrence. This increase was more pronounced with recurrent stone formation, indicating a close relationship between *Escherichia-Shigella* and both stone formation and recurrence, which aligns with earlier research findings [32, 33]. Other taxa implicated in the recurrence of stones include *Pseudomonadota, Gammaproteobacteria, Enterobacteriales* and *Enterobacteraceae*, which exhibited significant differences between the RS group and the FS group, while no notable differences were found between the FS group and the NS group. This suggests that an increased relative abundance of these bacteria is associated with stone recurrence. Similarly, the abundance of *Bacilliota* and *Clostridia* decreased in the RS group, indicating their potential role in stone recurrence. In contrast, *Oscillospirales* and *Subdoligulum* exhibited decreased abundance in both RS and FS groups, suggesting their involvement in stone formation while remaining stable during subsequent recurrences. Many studies have shown that the incidence rate of kidney stones in men is higher than that in women. Our diversity analysis suggests that the diversity and evenness of male gut microbiota are lower than those of female patients. Concurrently, the extent of gut microbiota dysregulation in male stone patients was generally more severe than in female patients. Previous studies have reported an increase in the levels of *Lachnospiraceae, Prevotellaceae* and *Peptostreptococaceae* in male urinary metabolism [7]. Conversely, *Muribaculaceae, Clostridiaceae, Clostridium-*sensu*-stricto, Clostridiales, Negativibacillus* and *Desulfovibrio* are more abundant in the female gut microbiota. We observed that most of the gut microbiota enriched in the intestines of female stone patients are associated with the development of intestinal inflammation [34, 35].

Denburg's research demonstrated no significant differences in the gene abundances of frc and oxc between stone-forming and healthy populations [8, 9]. Our study corroborated these findings and further revealed no significant changes in these key enzymes even with recurrent stone formers. However, we observed differential expression of oxdc exclusively between the RS group and the FS group. Oxdc was once regarded as a promising candidate for genetically regulating calcium oxalate stones [36]. Its high activity in *Bacillus* subtilis [37] aligns with our 16S rDNA sequencing results showing significant enrichment of *Bacillus* in the RS group. Recurrent kidney stones may indicate persistent or exacerbated oxalate metabolism disorders. Elevated oxalate levels likely induce oxdc overexpression to degrade excess oxalate. A double-blind clinical trial by Langman [38] demonstrated that oral administration of a recombinant, oxalate specific, microbial enzyme (oxdc) significantly reduced urinary oxalate excretion by degrading dietary oxalate in the gastrointestinal tract, offering therapeutic potential for hyperoxaluria and stone recurrence.

Untargeted metabolomics showed enrichment of Purine and Caffeine metabolic pathways. Three recent large cohort studies totaling more than 250,000 patients demonstrated that caffeine and its metabolites significantly inhibited calcium oxalate stone formation [39]. Caffeine is catabolized into three primary products: theobromine, theophylline and paraxanthine by CYP1A2 and CYP2E1, and untargeted metabolomics suggests that in the RS group, the primary metabolite of caffeine—paraxanthine (FC = 0.39, *p* = 0.0255) and the final metabolite–xanthine (FC = 0.52, *p* = 0.0005), were significantly less than those in the FS group. We have previously examined differences in metabolites in the urine of patients with stones and healthy populations and found significant differences in caffeine-related metabolites 7-methylxanthine, 7-methyluric acid, paraxanthine and 3,7-dimethyluric acid, all of which showed a consistent trend toward a decrease in the stone patients. The enriched pathways were mainly concentrated in 8 metabolic pathways including caffeine, phenylalanine and tryptophan [40]. This is in general agreement with our differences in metabolic pathways and metabolites in the intestinal flora of patients with recurrent and primary kidney stones.

As early as 2020, Toydemir reported that caffeine upregulates most Nrf2 pathway genes in Caco-2 cells [41]. Kanlaya further revealed that caffeine exerts anti-inflammatory effects by activating the Nrf2 signaling and inhibiting the Snail1 transcription factor. [42] These in vitro findings offer a potential biological mechanism for the observed association between caffeine metabolism and stone recurrence in our study, but further rigorous experiments are needed to confirm its functional involvement in calcium oxalate stone recurrence [43]. The downregulation of caffeine metabolism in the RS group may be correlated with the enrichment of *Bacillus* or *Escherichia-Shigella*. It is hypothesized that some *Bacillia* may secrete P450-like enzymes that compete with CYP1A2 for substrates (e.g., caffeine), potentially altering metabolic efficiency—but this remains speculative and requires experimental validation [44, 45]. Overproliferation of *E. coli* may lead to LPS entry into the bloodstream, activating liver macrophages (Kupffer cells), releasing cytokines such as TNF-α and IL-6, and inhibiting CYP1A2 activity (clinical studies have shown that CYP1A2-mediated drug metabolism is reduced in inflammatory states) [46–48]. Unfortunately, however, the metagenomic sequencing has not been enriched for the genes of CYP1A2 and CYP2E1, and our speculation would require further validation.

Although the phenylalanine, tyrosine and tryptophan biosynthesis pathway showed initial differences in untargeted metabolomics analysis, these differences did not retain statistical significance after FDR correction. Tryptophan, an essential amino acid, is primarily metabolized via the kynurenine pathway, generating metabolites such as kynurenine, kynurenic acid and quinolinic acid (QA) [49]. Previous studies demonstrated that QA induces renal resident cells to secrete pro-inflammatory cytokines (e.g., IL-1β, IL-6) by activating the NLRP3 inflammasome, triggering local inflammation linked to kidney stone formation [50, 51]. Our prior work revealed crosstalk between caffeine and aromatic amino acid metabolism [40].

We must recognize that our study has some limitations. First, while we employed a comprehensive matching strategy to balance key confounders, we acknowledge that PSM could further adjust for residual confounding. However, given the exploratory nature of this multi-omics study and effective baseline balance, PSM was unnecessary. Future confirmatory studies with larger cohorts may consider PSM to strengthen causal inference. Second, pre-surgical sample collection prevented confirming the temporal sequence between flora/metabolite differences and stone occurrence, requiring causal relationship validation. Third, unvalidated factors linked to kidney stone recurrence need verification via cellular or animal experiments. Additionally, we used PICRUSt2 for functional prediction, which infers entire genomes from a small hypervariable region of the 16S rRNA gene and may thus introduce inherent inaccuracies. Fourth, we excluded major factors interfering with gut microbiota during enrollment and follow-up, including antibiotic use, dietary changes, gastrointestinal diseases, hypertension, diabetes and malignancies. However, unrecognized or unmeasured confounders may still impact gut microbiota composition and metabolism. As kidney stone-related microbiota and metabolomics are influenced by multiple factors, these unforeseen variables could affect the robustness of our findings.

## Conclusions

This multi-omics study reveals that first-episode nephrolithiasis patients exhibit reduced gut microbial diversity compared with healthy individuals, while recurrent cases are characterized by distinct gut microbial community structure and evenness dysregulation. Recurrent patients show enrichment of *Enterobacterales, Pseudomonadota, Gammaproteobacteria, Enterobacteraceae, Escherichia-Shigella* and *Bacillia*, alongside dysregulated metabolic pathways including purine metabolism, and caffeine metabolism. Notably, caffeine and its metabolites such as paraxanthine and xanthine are significantly downregulated in recurrent patients, suggesting a potential association with inhibiting stone progression that merits further investigation. Ten metabolites including Asn-Tyr, Leu-Ala-Ile and 3'-hydroxyhexobarbital show promise as recurrence biomarkers, with four of them exhibiting significant correlations with *Escherichia-Shigella* abundance. Gender-specific gut microbial signatures may contribute to disparities in kidney stone susceptibility between the two sexes. Collectively, these findings highlight the critical role of the gut microbiome-metabolome axis in renal stone recurrence and provide novel microbial and metabolic targets for the early prediction, prevention and personalized treatment of this condition.

## Supplementary Information

Supplementary material 1.

## Data Availability

All raw sequencing data generated in this study have been deposited in the NCBI Sequence Read Archive (SRA) under the BioProject accession number PRJNA1404614 (https://www.ncbi.nlm.nih.gov/bioproject/PRJNA1404614). All untargeted metabolomic data used in this publication have been deposited to the EMBL-EBI MetaboLights database with the identifier MTBLS13960 (https://www.ebi.ac.uk/metabolights/MTBLS13960).

## Abbreviations

LC–MS: Liquid chromatography-mass spectrometry
RS group: The recurrent kidney stone group
FS group: The first-episode kidney stone group
NS group: The non-stone group
ASV: Amplicon Sequence Variants
KEGG: Kyoto Encyclopedia of Genes and Genomes
COG: Clusters of Orthologous Groups
QC: Quality control
PLS-DA: Partial least squares discriminant analysis
VIP: Variable importance in projection
FC: Fold change
PCA: Principal component analysis
NMDS: Non-metric multidimensional scaling
ANOSIM: Analysis of similarities
LEfSe: Linear discriminant analysis effect size
IQR: Interquartile range
ANOVA: Analysis of Variance
BMI: Body mass index
USG: Urinary specific gravity
Scr: Serum creatinine
OxlT: Oxalate: formate antiporter
Frc: Formyl-CoA transferase
Oxc: Oxalyl-CoA decarboxylase
Oxdc: Oxalate decarboxylase
DEMs: Differentially expressed metabolites
SCFAs: Short-chain fatty acids
QA: Quinolinic acid
