# Supplementary material for: Gut microbiome and metabolome signatures in calcium oxalate stone recurrence: a multi-omics study
Source: Microb Cell Fact. 2026 Mar 10;25:100. doi: 10.1186/s12934-026-02977-0 (PMC13088856; doi:10.1186/s12934-026-02977-0)
Supplement: Supplementary file 1 — Supplementary material 1. [file 12934_2026_2977_MOESM1_ESM.pdf]

Supplement information

Enrollment criteria:

（1）Both genders, aged 18-75 years, who provided informed consent.

（2）Individuals living long-term in Beijing, Tianjin, Hebei, or surrounding areas.

Exclusion criteria:

（1）Patients with other gastrointestinal diseases or previous gastrointestinal surgeries (e.g., inflammatory bowel disease, intestinal infections, irritable bowel syndrome).

（2）Patients who have used antibiotics in the last three months.

（3） Patients were excluded if they had extreme daily water intake (<1000 mL or >3000 mL), skipped breakfast ≥5 times/week, excessively consumed coffee/strong tea (≥4 servings/day or ≥28 servings/week), adhered to a strict vegan diet for ≥6 months, or had a significantly unbalanced dietary structure (e.g., abnormal intake of high-oxalate/high-purine foods, staple foods, fruits or vegetables).

（4）Individuals with other chronic diseases (e.g., chronic kidney disease, hypertension, diabetes, obesity [BMI > 28], or a history of tumors).

（5）Patients unable to eat normally.

（6）Patients with acute or chronic kidney disease.

（7）Patients who have used antibiotics or probiotics in the past three months.

Table S1 Stone composition distribution in the RS group and FS group

Kidney stone types	RS group (n=37)	FS group (n=38)
Pure calcium oxalate (monohydrate)	19（51.35%）	21（55.26%）
Pure calcium oxalate (dihydrate)	13（35.14%）	13（34.21%）
Mixed calcium oxalate + calcium phosphate	3（8.11%）	2（5.26%）
Mixed calcium oxalate + carbonate apatite	2（5.40%）	2（5.26%）
Urate stones, cysteine stones, and other types of stones	0	0

Distribution of kidney stone compositions in patients with the RS and FS groups. Data are presented as number (percentage) [n (%)].

Table S2 P-value table of clinical indicators with statistically significant differences.

P value	RS vs FS	RS vs NS	FS vs NS
Serum Uric Acid	0.116#	0.433#	**0.002**#
P	1.000*	0.124*	**0.010***
Family History	0.124※	**0.020**※	0.968※
Urine PH	**0.007***	0.243*	0.548*
USG	0.073*	**0.026***	**0.001***

Bold values indicate significant differences. USG, urinary specific gravity; Comparisons were made using Kruskal-Wallis test(*), Analysis of Variance(ANOVA,#) and 2-sided Chi-square test(※) Abbreviations: RS = Recurrent stone group, FS = First-espode stone group, NS = Non-stone group, USG = Urinary specific gravity.

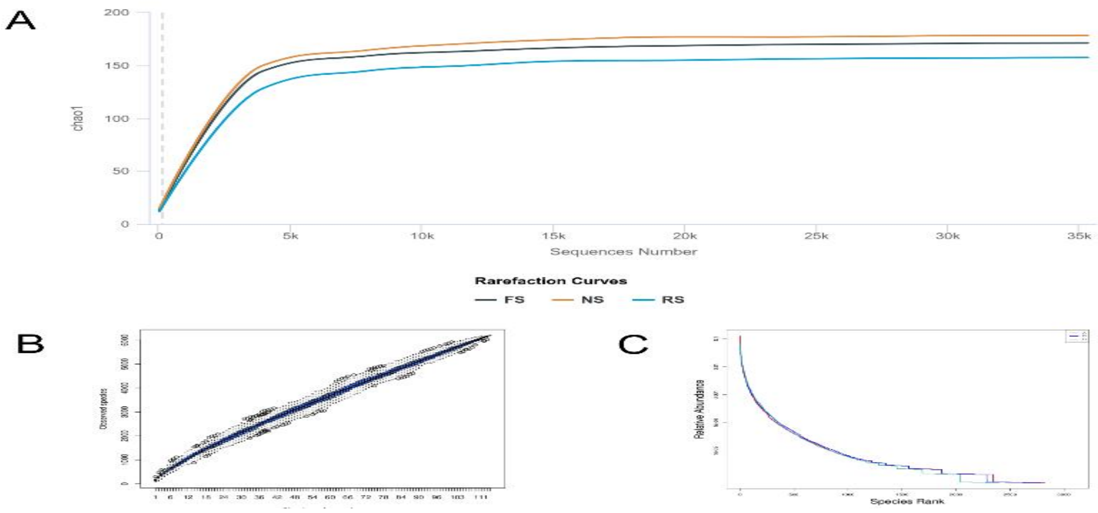

Fig. S1 Abundance curves estimating sample evenness and richness. (**A**) The rarefaction curves. (**B**)The rank-abundance curves. (**C**)The species accumulation curves.

Table S3 Comparison of gut microbial α-diversity indices among the RS, FS and NS groups

P value	RS vs FS	RS vs NS	FS vs NS
simpson	0.259	**7.000×10^{-4}**	**0.021**
shannon	0.067	**1.000×10^{-4}**	**0.039**
pielou_e	0.156	**2.000×10^{-4}**	**0.015**
dominance	0.259	**7.000×10^{-4}**	**0.025**
chao1	0.479	0.164	0.490
observed_features	0.458	0.146	0.473
goods_coverage	0.677	0.260	0.121

Comparison of gut microbial α-diversity indices among the RS, FS and NS groups. α-diversity indices include Simpson, Shannon, Pielou_e, Dominance, Chao1, Observed_features, and Goods_coverage. Comparisons were made using Kruskal-Wallis test. Data are presented as P-values for pairwise comparisons between groups. Abbreviations: RS = Recurrent Stone Group, FS = First-onset Stone Group, NS = Healthy Control Group

Table S4 Analysis of similarities (ANOSIM) test.

Group	R-value	P-value
RS-FS	0.03	0.05
RS-NS	0.1	0.001
FS-NS	0.04	0.02

The R value ranges between (-1,1). An R value > 0 indicates significant inter-group differences, while an R value < 0 suggests intra-group differences exceed inter-group differences. Statistical reliability is indicated by the P-value, where P < 0.05 denotes statistical significance.

Table S5 Demographic characteristics of metagenomic-selected and remaining patients in

each group.

Variables	RS group			FS group			NS group		
	n=27	n=10	p	n=28	n=10	p	n=29	n=10	p
Age	46.00 (36.00, 58.00)	52.00 (37.75, 54.75)	0.549	45.50 (39.75, 51.00)	45.50 (42.75, 50.25)	0.894	46.00 (38.00, 55.50)	55.00 (36.25, 59.50)	0.367
Gender			0.445			0.449			1.000
Male	17(63.00)	8(80.00)		19(67.86)	5(50.00)		19(65.52)	6(60.00)	
Female	10(37.00)	2(20.00)		9(32.14)	5(50.00)		10(34.48)	4(40.00)	
BMI	26.51 (23.67, 28.34)	26.11 (23.71, 31.53)	0.851	25.95 (23.27, 26.81)	26.35 (24.55, 30.98)	0.289	25.26 (24.16, 27.34)	24.67 (23.40, 26.34)	0.288
Family History			0.360			1.000			1.000
Yes	3(11.11)	3(30.00)		2(7.14)	1(10.00)		2(6.90)	1(10.00)	
No	24(88.89)	7(70.00)		26(92.86)	9(90.00)		27(93.10)	9(90.00)	
Operating Time	67.00 (39.00, 85.00)	70.00 (50.00, 97.00)	0.462	49.00 (38.50, 78.75)	52.50 (28.75, 76.25)	0.496	NA	NA	NA
Average CT Value	643.00 (410.00, 793.00)	478.50 (376.75, 594.65)	0.182	508.00 (353.25, 771.25)	409.50 (270.25, 534.50)	0.239	NA	NA	NA
Serum Uric Acid	407.60 (326.70, 463.00)	421.60 (374.95, 453.00)	0.494	361.60 (288.35, 419.18)	392.05 (283.93, 426.85)	0.666	327.80 (259.90, 386.20)	329.36 (264.25, 359.35)	0.573
P	1.12 (1.02, 1.21)	1.09 (0.95, 1.30)	0.973	1.16 (1.06, 1.29)	1.08 (0.89, 1.29)	0.361	1.23 (1.10, 1.34)	1.31 (1.14, 1.35)	0.334
Ca	2.31 (2.30, 2.34)	2.28 (2.22, 2.33)	0.538	2.28 (2.23, 2.33)	2.34 (2.24, 2.36)	0.239	2.35 (2.23, 2.42)	2.34 (2.24, 2.42)	0.910
Scr	78.60 (65.20, 99.30)	78.05 (70.90, 93.48)	0.719	79.85 (65.15, 99.98)	76.35 (48.78, 90.58)	0.304	73.20 (57.95, 80.30)	70.20 (62.58, 84.25)	0.962
Urine PH	6.00 (6.00, 7.00)	6.00 (6.00, 7.00)	0.635	6.00 (5.50, 6.38)	6.00 (5.50, 6.13)	0.740	6.00 (6.00, 7.00)	6.00 (6.00, 6.25)	0.905
USG	1.02 (1.01, 1.02)	1.02 (1.01, 1.02)	0.582	1.02 (1.02, 1.02)	1.01 (1.01, 1.02)	0.244	1.02 (1.02, 1.02)	1.02 (1.02, 1.03)	0.699

①Data are presented as median (25th percentile, 75th percentile) [M (Q25, Q75)] for continuous variables and number (percentage) [n (%)] for categorical variables.

②For continuous variables with non-normal distribution (including age, BMI, operation time, mean CT value, serum phosphorus, serum calcium, serum creatinine, urine pH, and urine specific gravity), comparisons between groups were performed using the Mann-Whitney U test; For categorical variables (gender and family history), comparisons between groups were conducted using the Fisher's exact test

③The significance level was set at α=0.05. A P-value > 0.05 indicates no statistically significant difference, while P ≤ 0.05 indicates a statistically significant difference.

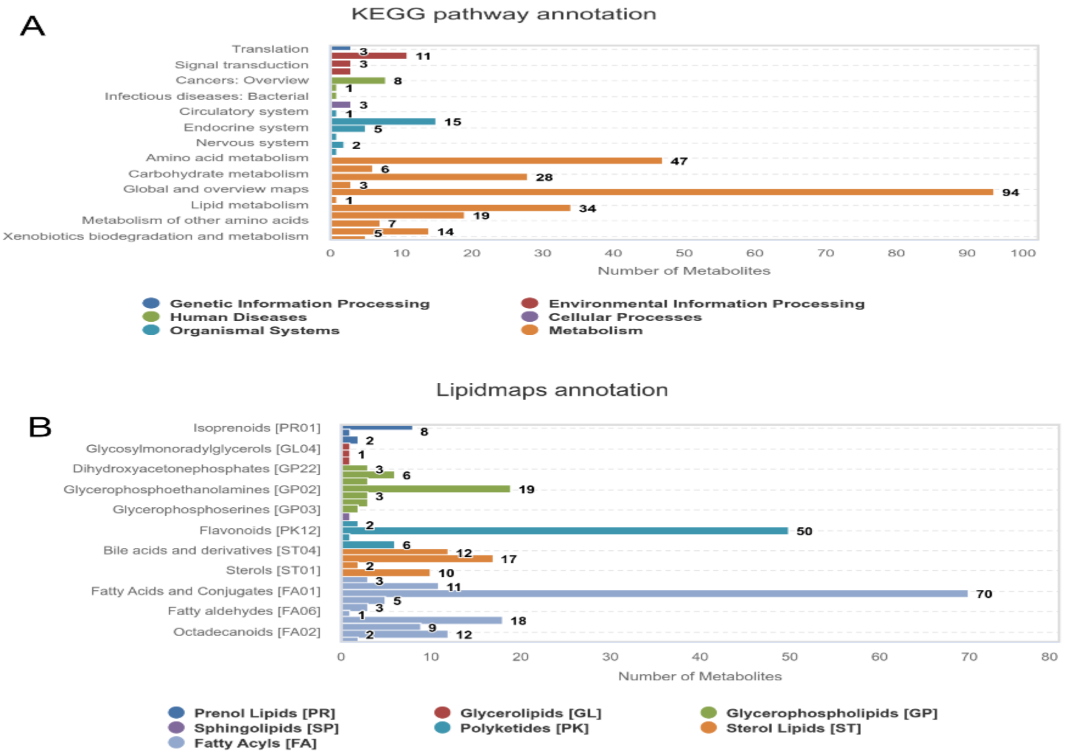

Fig. S2 Metabolites annotation by KEGG and Lipidmaps. (**A**) Metabolite distribution of KEGG pathway annotation under anion mode. (**B**) Distribution of lipid metabolites annotated by Lipidmaps under anion mode.
